# Enhancing Solid State LiDAR Mapping with a 2D Spinning LiDAR in Urban Scenario SLAM on Ground Vehicles

**DOI:** 10.3390/s21051773

**Published:** 2021-03-04

**Authors:** Weichen Wei, Bijan Shirinzadeh, Rohan Nowell, Mohammadali Ghafarian, Mohamed M. A. Ammar, Tianyao Shen

**Affiliations:** Robotics and Mechatronics Research Laboratory, Department of Mechanical and Aerospace Engineering, Monash University, Clayton, VIC 3800, Australia; bijan.shirinzadeh@monash.edu (B.S.); rohan.nowell2@monash.edu (R.N.); mohammadali.ghafarian@monash.edu (M.G.); Mohamed.Ammar@monash.edu (M.M.A.A.); tianyao.shen@monash.edu (T.S.)

**Keywords:** SLAM, LiDAR, localisation, UGV

## Abstract

Solid-State LiDAR (SSL) takes an increasing share of the LiDAR market. Compared with traditional spinning LiDAR, SSLs are more compact, energy-efficient and cost-effective. Generally, the current study of SSL mapping is limited to adapting existing SLAM algorithms to an SSL sensor. However, compared with spinning LiDARs, SSLs are different in terms of their irregular scan patterns and limited FOV. Directly applying existing SLAM approaches on them often increase the instability of a mapping process. This study proposes a systematic design, which consists of a dual-LiDAR mapping system and a three DOF interpolated six DOF odometry. For dual-LiDAR mapping, this work uses a 2D LiDAR to enhance a 3D SSL performance on a ground vehicle platform. The proposed system takes a 2D LiDAR to preprocess the scanning field into a number of feature sections according to the curvatures on the 2D fraction. Subsequently, this section information is passed to 3D SSL for direction feature selection. Additionally, this work proposes an odometry interpolation method which uses both LiDARs to generate two separated odometries. The proposed odometry interpolation method selectively determines the appropriate odometry information to update the system state under challenging conditions. Experiments are conducted in different scenarios. The results proves that the proposed approach is able to utilise 12 times more corner features from the environment than the comparied method, thus results in a demonstrable improvement in its absolute position error.

## 1. Introduction

Solid-State LiDAR (SSL) overcomes many limitations of spinning 3D LiDARs, such as high cost and manual tuning. Mainly manufactured using MEMS, SSLs are more compact, flexible and low-budget than their competitors. However, the majority of the SSLs are restricted by Field-of-View (FOV). SSLs does not use a traditional rotary structure. Instead, their optical components are often directional, which provide the SSL with a narrow FOV. Limited FOV significantly restricts the application of SSLs, especially on autonomous vehicles, where fast scanning rate and broad coverage are still the two most demanding features.

Furthermore, unlike spinning LiDARs, the laser beam from SSL does not necessarily move in circles. Instead, many SSLs have irregular scan pattern. For example, the Livox Mid series’s laser beam follows a petal shape scan pattern which symmetrically covers the FOV. Table 1 shown the performance comparison between a 2D spinning LiDAR Hokuyo UST-20LX, a SSL Livox Mid-40, and a multi-line 3D spinning LiDAR Velodyne HDL-64E. Compared with traditional spinning LiDAR, using the irregular scan pattern of SSLs has two major concerns. The first problem is that Livox Mid series generate 3D points via a single laser beam, which is much slower than multi-beam spinning LiDARs. According to its specification, Livox Mid-40 takes 1 s to cover 95% of its FOV. Secondly, since the scan pattern is in rotating petal shape, the laser beam will not cover the same surface in the previous scans. The non-repetitive scan path challenges a mapping system to identify a feature between two consecutive scans.

The highly cost-efficient SSLs rapidly increased its market share in the past few years. While many researchers tried to address the above problems directly by strengthening the accuracy of the scan-matching algorithms, this problem could be improved through other aspects of a SLAM system.

This work presents a 2D and 3D hybrid LiDAR mapping system which aims to address the limitations of a single SSL mapping unit. In the design, the mapping system features a 2D horizontal LiDAR and a 3D forward-facing SSL. The mapping system is mounted on top of a ground vehicle to perform mapping tasks. A dual-LiDAR cooperative model is presented which improves the accuracy and robustness of the hybrid mapping system. The major contributions of this paper are as follows:

- Two-Stage Point Cloud Processing. This work proposes a two-step point cloud process which uses the 2D LiDAR readings to preprocess the scanning field of the 3D LiDAR. By segment the FOV in to sections with feature labels, the proposed two-step feature extraction process provides a more targeted feature point extraction approach to the SLAM system.
- Three DOF Interpolated six DOF Odometry. The proposed dual-LiDAR odometry uses the additional three DOF (*x*, *y* and yaw) odometry generated by the 2D LiDAR to stabilise the six DOF odometry estimation from the 3D SSL measurements.
- High Cost-Efficiency 3D SLAM System. While the proposed system features a 2D LiDAR and a 3D SSL, the setup developed in this work only costs a fraction of a multi-line spinning LiDAR. Combining the two LiDARs enables the system to have a semi-omnidirectional vision of the scanning environment, thus utilising the surrounding environment features.

The system proposed in this work can be described in three parts: the algorithm design, the software architecture and the hardware setup. The following sections first review some of the existing approaches, including some state-of-the-art 3D LiDAR mapping algorithms and methods specialised for SSLs. This is then followed by the explanation of the proposed system, including the point cloud segmentation, scan-matching process and odometry interpolation. After that, the experiments used to evaluate the proposed system are presented, including a detailed explanation of the hardware setup. Different sets of experiments were conducted on various places on the Monash University campus, which demonstrated the system’s behaviour in different environments. Last but not least, the final part of this paper concludes the contributions of the proposed system and discusses some limitations that could be improved.

## 2. Multi-LiDAR Mapping Overview

LiDAR SLAM has rapidly developed over recent years. The mapping technology quickly evolved from 2D localisation to 3D Mapping [1,2,3,4,5,6,7]. Similar to other SLAM approaches, LiDAR SLAM algorithms largely depend on the type of sensors. To enhance the performance and overcome the shortcomings of the single LiDAR mapping system, many researchers took efforts to innovate the design of a LiDAR mapping system. These efforts include incorporating multiple LiDARs into a single mapping unit, extend the motion range of a single LiDAR sensor and reengineer the mechanical feature of a LiDAR sensor. This section reviews some of the recent achievements in this field that are related to the design of the proposed system.

### 2.1. Multiple LiDAR Cooperation in SLAM Systems

Combining multiple LiDARs in a mapping unit often aims at enhancing the performance of a SLAM system. Despite the fact that multi-LiDAR system requires extra efforts to merge the readings before processing, adding an extra LiDAR to the system directly enlarges the FOV of the sensing unit. In most of the multi-LiDAR systems, LiDARs are horizontally aligned to ensure the same scanning direction [8,9,10]. In these works, the number of LiDARs directly amplifies the scanning field. An FOV enhancing approach uses five LiDARs mounted on each side of a car, with their scan direction parallel to the ground [10]. It uses 16-line LiDARs to perform objection in a merged point cloud. Existing studies emphasise merging multi-LiDARs to generate an enormous point cloud, which requires calibration during initialisation.

Calibration of multiple LiDARs aims at finding the transformation matrix between the LiDARs and the robotic system odometry. Studies in this field use reflective conic items which appear in both LiDAR scan results to calculate the displacement and rotation between them [11]. Checkerboard calibration methods are also effective for calibrating mixed types of sensors [12,13,14]. Changing the shape and pattern on the calibration board allows different sensors to identify their transformation matries to the checkerboard, and thus with respect to other system components. Other than a checkerboard, researchers use a spherical shape item to calibrate both cameras and LiDARs on an autonomous vehicle [15]. However, the sensor position is constantly changing in real-world scenarios. There are some calibration methods which do not relying on a pre-known target. Using Iterative Closest Points (ICP) algorithms [16,17,18,19] to calculate the geometrical relationship between two sets of point clouds only requires the LiDARs to have overlapped scanning areas [20,21]. When processing high-frequency LiDAR readings, synchronisation of the readings and minimisation of the time gaps between the LiDARs are critical to the merging process. Researchers have discussed the relationship of timestamps and synchronisation in multi-LiDAR calibration [22,23].

Instead of finding the nearest neighbour between two sets of point clouds, some approaches only compare the trajectories generated from different sensors to identify the transformation between them, which significantly reduces the computational complexity of the calibration [24,25].

### 2.2. Cross-Dimensional Feature Extraction from LiDAR Data

Unlike RGB cameras, which use CMOS to generate a 2D pixel matrix, LiDARs use a moving laser beam to sample the environment. The mechanical nature of the LiDAR sensors makes the output point cloud contain strong geometrical information. Researches have used this geometrical feature of the LiDAR sensor to create high-dimensional images from low-dimensional LiDARs [26,27,28]. Using a 2D LiDAR attached to the top of a voice coil helps the LiDAR to perform z-axis motions [27]. A 10 mm displacement generated from the voice coil allows the system to produce 2.5D maps with only a 2D LiDAR. Instead of linear motion on the z-axis, rotating along the x-axis is also a common approach to produce 3D reading from 2D LiDARs [26,29]. Pfrunder et al. [30] used an inclined 2D LiDAR to scan through space with the six DOF motion of the ground vehicle recorded by other sensors. Similar approaches can also be seen in other works [31,32].

Compressing 3D point clouds into a lower-dimensional format improves the transmission and storage of the generated map [33,34,35]. The feature compressing methods significantly improved the mapping system’s performance in the urban environment for two reasons. Firstly, urban synthetic scenes are often perpendicular to the ground [36,37,38]. Thus, downgrading the 3D map into 2D ‘bird’s-eye view’ maps has little effect on the navigation system [34]. Secondly, the compressed data stream improves the connectivity of a robotic system in the network [33].

### 2.3. SLAM Systems with Solid-State LiDAR

Spinning multi-line LiDARs occupy a large share of the LiDAR market, both in research and industry. The spinning mechanism ensures the laser beam repeatedly covers the same area in different scans. However, in recent years, solid-state LiDAR (SSL) become a rising power on the market. Compare with traditional spinning LiDAR, SSLs have less moving parts, more compact design, low power consumption and higher reliability [39]. More importantly, SSLs generally cost less than spinning multi-line LiDARs [40].

While SSL development seems to have a promising future, directly applying spinning LiDAR algorithms on them can be difficult. Most SSLs have irregular scan patterns, such as ‘Z’ shape, petal shape or ellipse shape. Researchers have re-engineered the feature matching algorithms to adapt different kinds of scan patterns [41,42].

Additionally, SSLs often have lower sampling rates compared with traditional spinning LiDAR. With a rotating motor, a spinning LiDAR can easily maintain its scan frequency above 20 Hz. However, many SSLs can only provide 10 to 15 Hz scan frequency, which requires more robust motion blur methods [39].

Furthermore, since most of the SSLs are manufactured with Micro-electromechanical Systems (MEMS), the optical mechanism restricted their field-of-view (FOV) [43]. Using Velodyne Velarray M1600 as an example, the sensor only has a 120° horizontal FOV and 35° vertical FOV. Without fusion with other sensors, such a limited rectangle shape view window limits the performance of the SLAM system [44]. Lin and Zhang [42] improved this problem by using extra information for feature matching. Besides depth information, this work also uses intensity as the supplemental data for feature matching.

## 3. Dual-LiDAR Mapping Unit Setup

**Figure 1:**
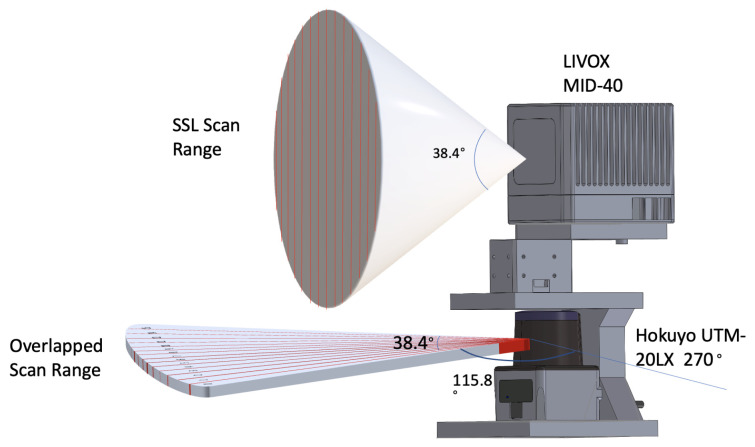
Physical layout of the mapping unit.

To overcome the limitations of using single SSL as the LiDAR SLAM system’s input, this section describes a 2D-3D hybrid dual-LiDAR mapping unit. The proposed mapping unit uses Hokuyo UST-20LX, which was purchased from Hokuyo, Osaka, Japan, as the 2D LiDAR and Livox Mid-40, which was purchased from Livox Technology, Shenzhen, China, as the 3D LiDAR. Figure 1 shows the layout of the system. The x- y- and z-axis indicate the Front-Left-Up (FLU) of the coordinate system in presented work. Under FLU, the two LiDAR are vertically aligned with the positive direction of their x-axes facing the fount of the mapping system. The two LiDARs are manually calibrated using the method described in [11,13], such that, the shape of a reflective tape can be identified in LiDAR readings using their intensity measurements. A 1 m long, 1 cm wide, horizontally aligned reflective tape was carefully placed on a flat surface and adjusted using a laser interferometer-based tracker. The Hokuyo UST-20LX was installed by aligning its reading with the reflective tape. Using the Hokuyo UST-20LX as as the reference, the relative position of the livox Mid-40 can be obtained via the matching the reflective tape in two LiDAR readings using Iterative Closest Point (ICP).

Livox Mid-40 offers a 38.4° FOV in a 3D cone shape. On contrast, Hokuyo UST-20LX receives 2D laser readings in 270°. The two sensors have an overlapped FOV in the middle of their scan range where the Hokuyo observes a 2D fraction of the Livox scan field (Figure 1).

## 4. Point Cloud Preprocessing under the Manhattan-World Assumption

**Figure 2:**
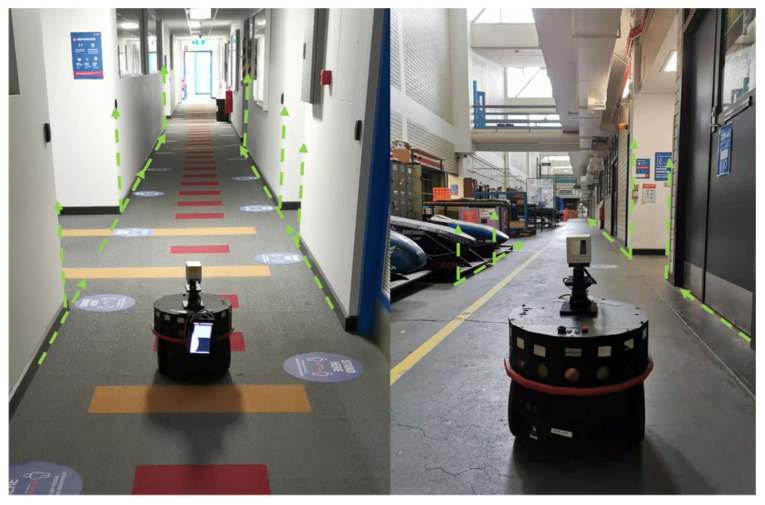
Corridors in the engineering department at Monash University with the wall and corner features vertically aligned.

As illustrated in Figure 1, the 2D LiDAR scan has a 38.4° overlapping FOV with the 3D LiDAR reading. The proposed study uses the 2D LiDAR reading which falls under this range to preprocess the 3D LiDAR scan. The proposed system is designed for unmanned ground vehicles (UGV) in an urban scenario, which allows this study to hypothesise that the 2D LiDAR is horizontally aligned with the scanning environment. Under the Manhattan-World Assumption [37], the corners and planes in synthetic scenes exhibit a strong geometrical relationship. This is especially the case in the constructed area where walls and corners are often in an axis-aligned convention. Figure 2 shows the layout of the corridor in Engineering Building on Monash University campus. In the picture, the corner and surface features are axes-aligned. Vertically splitting the scanning field helps to isolate corner or planes from the scan result.

### 4.1. 2D Section Selection

With the Manhattan-World Assumption, it is believed that the plane and corner features observed with the 2D LiDAR are likely to be repeated vertically in the 3D LiDAR reading. The consistency of features on z-axis allows the system to use the 2D scan to pre-sample the 3D space.

**Figure 3:**
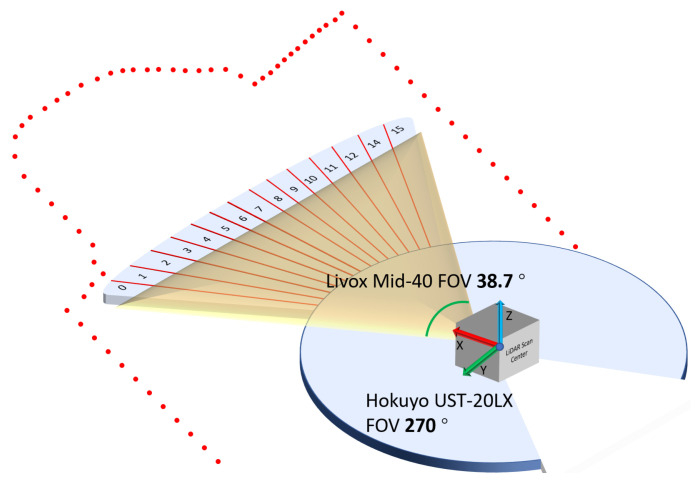
Overlapping FOV of the two LiDARs.

The preprocessing is achieved by calculating the curvature of the 2D reading in each sweep. Since this study is only interested in the 2D points overlapping in the 3D scan, unrelated points need to removed. Shown in Figure 3, the width of the FOV of Livox Mid-40 is 38.4°, whereas the FOV of the Hokuyo UST-20LX is 270°. Data from Hokuyo contains a distance reading *d* and a sequence number. Let $\phi_{a}$ be the incremental angle between each scan point and $s_{p}$ be the point sequence number.

(1) $$\phi_{a}=\frac{270^{\circ }}{s_{p}}$$

With the incremental angle of each point $\phi_{a}$ and distance reading *d*, the *x* and *y* coordinate of each LiDAR scan are:

(2) $$x=d_{p}*\cos\left(\phi_{a}*s_{p}\right)$$

(3) $$y=d_{p}*\sin\left(\phi_{a}*s_{p}\right)$$

Let *f* be the scale between the FOV of two LiDARs where

(4) $$f=\frac{38.4^{\circ }}{270^{\circ }}$$

Only 2D LiDAR points overlapped with the 3D LiDAR FOV are used in the section feature extraction. Calibration is required to align the FOV of the two sensors. This work uses the method described in [11,13] to obtain the transformation matrix between the Hokuyo UST-20LX and Livox Mid-40. Assuming each 2D LiDAR scan *S* has *m* points. After calibration, from 3D LiDAR’s coordinate frame, the point *p* from 2D LiDAR is selected for preprocessing if:

(5) $$\frac{m}{2}-\frac{f*m}{2}<=\phi_{a}*s_{p}<=\frac{m}{2}+\frac{f*m}{2}$$

Assuming there are *n* points between two points $p_{a}$ and $p_{c}$ in the scan *S*. Let $p_{b}$ be the middle point of this sweep section. The curvature of the scan section between $p_{a}$ and $p_{c}$, $\kappa_{p_{b}}$, can be described using Equation (6). Sorting all LiDAR measurements between $p_{a}$ and $p_{c}$ according to their curvatures provides the list of points with their curvatures in descending order.

(6) $$\kappa_{p_{b}}=\frac{1}{n\cdot \left|\right|p_{b}∥}∥\sum_{i\in n,i\neq b}\left(p_{b}-p_{i}\right)∥$$

With the setup shown in Figure 3, the FOV was divided vertically into 15 sections. These 15 sections include 10 corner sections and 5 plane sections. Correspondingly, the 2D scan plane is divided into 15 sections with 2.56° pre-section. With Hokuyo UST-20LX, there are about 10 scan points allocated in each section. The number of sections is selected base on the angular resolution of the 2D LiDAR equipped, the size of the overlapped FOV, and the environmental feature. The choice of selecting 10 corner sections and 5 plane sections is considered suitable for the testing scenarios proposed in the study.

Staring from the point with the highest curvature score, if the section of the selected point is not tagged yet, then mark the current section as a corner feature section. This process is repeated until there are ten corner sections selected. Similarly, five plane sections are marked based on the points have the smallest curvature value. This process is described in Algorithm 1. In this study, it was found that the number of both plane and corner sections can be changed to adapt to the application scenario.
**Algorithm 1** Mark Sections with Feature Tag.**Input:**$Points_{sorted}$; Section[] **Output:**Section[] with feature tag
    plane_number=5
    **for** (i=0,i<plane_number) **do**
        $S_{id}=\frac{\phi_{Points_{sorted}\left[i\right]}}{2.56}$        **if** $\left(Section\left[S_{id}\right]\;NOT\;marked\right)$ **then**            $Section[S_{id}]\;is\;plane\;section$        **else**            plane_number+=1        **end if**    **end for**
    corner_number=10
    **for** (i=0,i<corner_number) **do**
        $S_{id}=\frac{\phi_{Points_{sorted}\left[Points.Size-i\right]}}{2.56}$        **if** $\left(Section\left[S_{id}\right]\;NOT\;marked\right)$ **then**            $Section[S_{id}]\;is\;corner\;section$        **else**            corner_number+=1        **end if**    **end for**



### 4.2. 3D Feature Selection with 2D Preprocessing

3D feature selection in LOAM is based on the curvature of the scan line. While using a spinning LiDAR, the laser beams are travelling in a circle. The repeated trajectory lays the foundation for the feature selection method in most of the state-of-the-art SLAM systems. However, directly applying this method to the Livox Mid-40 in this project will face difficulties, including:

1. The petal shape scan path gives the scan trajectory a non-even curvature. This makes differentiating corners from planes more challenging.
2. The slow scanning rate results in a larger displacement for the same feature point appear in two consecutive scans, thus harder to be paired by the matching function.

**Figure 4:**
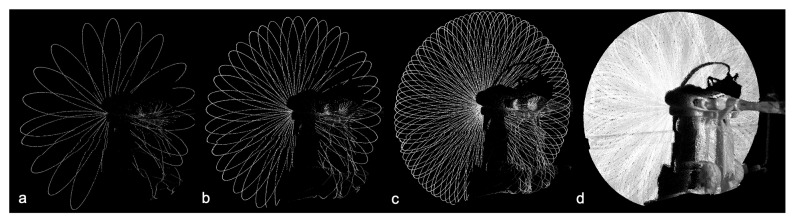
Points from Livox Mid-40 reading: (**a**) 100 ms, (**b**) 200 ms, (**c**) 1000 ms, (**d**) 5000 ms.

With intensity displayed in grayscale, Figure 4 shows a frame comparison of point cloud received from Livox Mid-40 within 100 ms, 200 ms, 1000 ms and 5000 ms, respectively. According to the specification of form Livox, the LiDAR only covers 20% of the FOV in 100 ms. Livox Mid-40 will need 1000 ms to cover 95% of the FOV. The irregular scan pattern makes the same spot takes a long time to be scanned twice. It is also worth noting the petal shape scan pattern created an uneven coverage of the scan FOV, which the centre of the FOV has a higher scan density than the edge.

**Figure 5:**
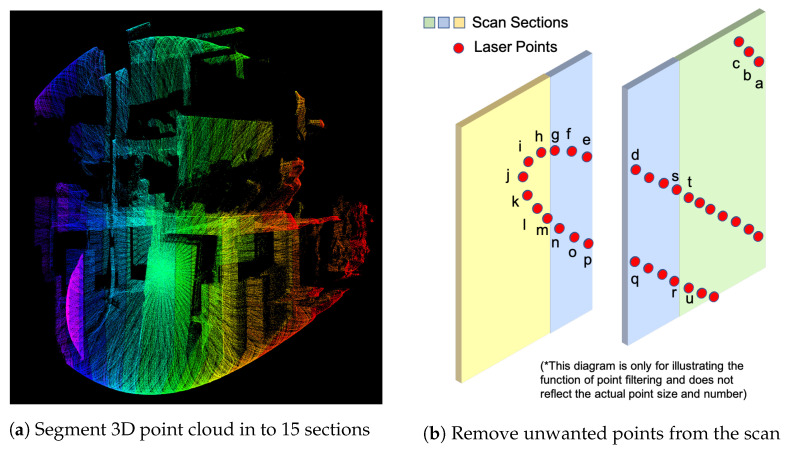
Scan sections and point filtering.

With section information from preprocessing in Section 4.1, the proposed system could segment the FOV into a number of sections with feature tags. The tags are indicating the most significant feature in the section, which provides the 3D SSL with an extra layer of information in point cloud processing. The results of vertically segmenting the 3D LiDAR readings into 15 sections is reflected in Figure 5a with different sections assigned with a different color.

The Livox reading was processed in a way similar to [29,42], where linear interpolation is used to restore each LiDAR reading from motion blur. The processed point cloud is then split into lines every half-a-petal. Under FLU, let $\theta_{p}$ be the angle between the 3D LiDAR point and the x-axis.

(7) $$\theta_{p}=\frac{arctan2(y,x)}{\pi }*180^{\circ }$$

Then the points on each lines were stored into the corresponding section container with sequence number $S_{id}$ according to:

(8) $$S_{id}=\frac{\theta_{p}}{2.56^{\circ }}$$

where $2.56^{\circ }$ is calculated by dividing $38.4^{\circ }$ FOV into 15 sections. With all LiDAR points been stored into section containers, the next step is to remove the unwanted points from the point cloud. Overall, four kinds of points are removed from the feature selection process:

- Scan pattern belongs to different sections are processed independently. Since it is difficult to estimate the curvature of a point on the end of a line, points close to the edge of each section are ignored. In Figure 5b, these points include: s,t,r,u,g,h,m,n.
- The fringe points on the edge of the LiDAR FOV are not considered for feature points due to the curved fringe beam path of the Livox Mid-40. The proposed methodology limited the FOV of the SSL to 37° to remove fringe points. In Figure 5b, these points include: k,j,i,h.
- When a corner is covered in a scan, the point on the far side of the LiDAR scan will be not considered as a feature point. Same as [29], it is considered that the far side of a corner point may not be visible in future scans. In Figure 5b, these points include: e,p.
- Since the 3D LiDAR scan is divided into sections, some scan lines only have a tiny intersection with a section. In the proposed method, a scan line with less than 6 points in a section will not be considered as candidature points in that section. In Figure 5b, these points include: a,b,c.

After removing unwanted points from the point cloud, the system performs feature selection from each section container. Each section container is attached with a feature type tag as described in Algorithm 1. This work only selects the tagged features from the corresponding section container.

The plane feature selection in 3D is similar to the 2D process, which is based on the curvature calculation in Equation (6). However, in the 3D point cloud, the number of neighbour points involved in the calculation was reduced. The point is considered a plane feature if the average curvature with its six nearest neighbours is less than 0.1.

On the other hand, the corner features are calculated differently. Let $L_{a}$ and $L_{b}$ be the two lines formed by the five nearest neighbours on each side of the target point $p_{c}$, respectively. Assume $\kappa_{L_{a}}$ and $\kappa_{L_{b}}$ be the curvatures of the two lines, where

(9) $$\kappa_{L_{a}}=\frac{1}{5\cdot \left|\right|p_{c}∥}∥\sum_{i=c-5}^{c}\left(p_{c}-p_{i}\right)∥$$

(10) $$\kappa_{L_{b}}=\frac{1}{5\cdot \left|\right|p_{c}∥}∥\sum_{i=c}^{c+5}\left(p_{c}-p_{i}\right)∥$$

Let $\theta_{c}$ be the angle between the two lines $L_{a}$ and $L_{b}$ normalised to the unit vector. The point $p_{c}$ is considered as a corner point where

$$\kappa_{a}<0.1\;and\;\kappa_{b}<0.1\;and\;70^{\circ }<\theta_{c}<120^{\circ }$$

**Figure 6:**
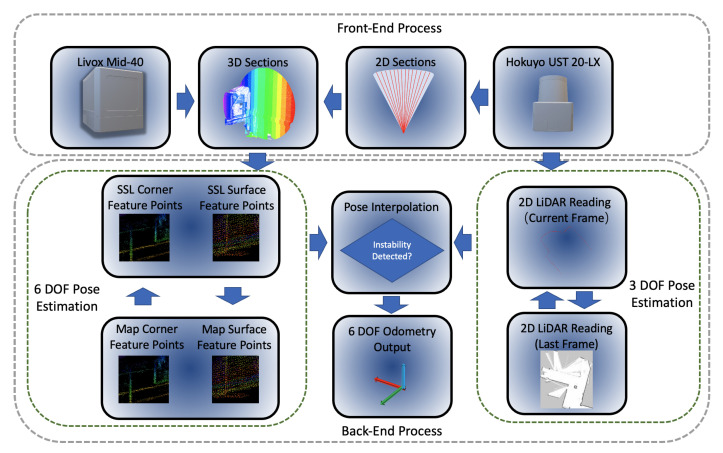
Data flow of the proposed dual-LiDAR odometry system.

With the selected feature points, a scan-matching is performed as in [42]. Figure 6 illustrates the data flow of point cloud processing with the readings from the Livox Mid-40 used in this work. Only corresponding feature points in the section are selected based on the tag type of the section. When only one feature is selected in each section, the proposed algorithm loosens the restriction of the number of points. Instead of 4 points on each scan line, maximally 1000 points are selected in each section. A VoxelGrid filter is applied to enhance the evenness of the sample feature points. The length of each edge of the voxel cube is set to 0.3 m. The choice of voxel size is made based on the environmental feature, the point cloud density, and the system performance.

## 5. Pose Estimation with Dual-LiDAR Sensing Unit

Single SSL SLAM systems suffer from their limited FOV. Fewer feature pairs in two consecutive scans cause the system to be sensitive to rapid movements, especially in sharp turns. To further strengthen the stability of the mapping system, this work also introduces a pose stabilisation mechanism that uses the pose estimation from the 2D LiDAR to stabilise the estimation from 3D LiDAR.

The calculation of the six DOF system pose is based on the plane and corner feature distance as in [42]. Besides the six DOF pose estimation, the proposed system also generates a three DOF incremental pose estimation on x-, y- and yaw-axis from the 2D LiDAR scan using Point-to-Line Iterative Closest Point (PL ICP). While the mapping system mainly relies on the six DOF estimation, the three DOF estimation provides a supplemental pose update.

The proposed design of dual-odometry targets the instability of the mapping system in extreme scenarios. As explained in Section 3, the FOV of the six DOF odometry is restricted to 38.4°, thus in the risk of insufficient feature points. Furthermore, the rapid change of the scan scenes will increase the difficulty of calculating the displacement between feature points. On the other hand, with Point-to-Line Iterative Closest Point (PL-ICP), calculating three DOF pose estimation based on 2D LiDAR readings provides a more stable and higher frequency odometry.

In the proposed work, the quality of the six DOF pose estimation is evaluated via two cost functions, which is in the same fashion of LOAM and Livox-LOAM. Let $p_{l}$ be a point in the LiDAR frame. After applying rotation and translation using the current LiDAR pose, the coordinates of $p_{l}$ in map frame is $p_{m}$. For a corner point, the Principal component analysis (PCA) is used to assure the nearest 5 neighbour points of $p_{m}$ on the map belongs to a corner feature where the biggest eigenvalue is three times lager than the second biggest eigenvalue. If the PCA process indicates the neighbours surrounding $p_{m}$ is forming a line, then Equation (11) is the residual function of the pose estimation.

(11) $$r_{corner}=\frac{\left|\left(P_{m}-P_{5}\right)\times \left(P_{m}-P_{1}\right)\right|}{\left|P_{5}-P_{1}\right|}$$

Similarly, if $p_{m}$ is a plane point, and the smallest eigenvalue of PCA of its 5 nearest neighbours is three times smaller than the second smallest eigenvalue, then $p_{m}$ is considered as a valid plane feature. Equation (12) is used for the pose estimation of plane features.

(12) $$r_{plane}=\frac{{\left(P_{w}-P_{1}\right)}^{T}\left(\left(P_{3}-P_{5}\right)\times \left(P_{3}-P_{1}\right)\right)}{\left|\left(P_{3}-P_{5}\right)\times \left(P_{3}-P_{1}\right)\right|}$$

On the other hand, the three DOF pose estimation from the 2D LiDAR uses PL-ICP, where the optimisation target is the minima squire error between current point and the normal vector of its two closest neighbours in the previous scan. Since the two LiDARs evaluated in this study have different publish rates, the 2D LiDAR scans used in three DOF pose estimation are recorded based on the frequency of 3D LiDAR measurements. The 2D LiDAR scans received between the 3D LiDAR frames are excluded from the pose estimation process.

The proposed approach keeps examining the two residuals from the six DOF pose estimation. If either of the preset thresholds are exceeded, the current six DOF pose update is replaced by the three DOF pose estimation transformed into the map frame. In this study, it was found that setting the threshold of plane feature residual to 0.01, and the corner feature residual threshold to 0.02 provide the most suitable outcome.

Upon updating the system state with three DOF (x-axis, y-axis and yaw) pose information, the z-axis, pitch and roll states are inherited from last system state. Noting that the proposed approach only modifies the three out of the total six DOF, which are the x-, y and yaw axes of the SLAM system. The UGV developed in this project can travel on a slope to generate motions in z-, roll and pitch axes, but the majority of motions, especially the sharp turns are more related to the x-, y- and yaw axes. Stabilising the motions in the modified three DOF provides the system with a more accurate six DOF system state for the next round of six DOF pose estimation, thus improves the six DOF pose estimation.

## 6. Hardware Design and Experiments

**Figure 7:**
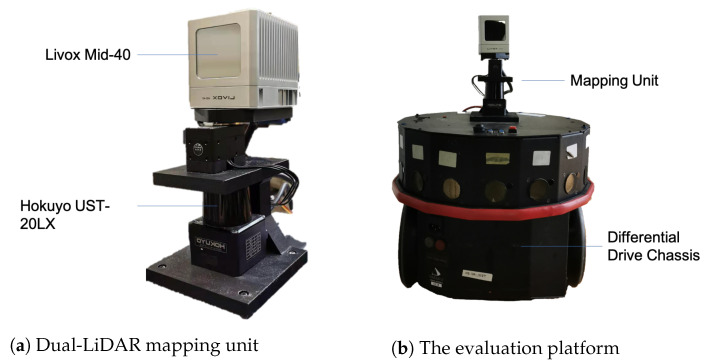
Developed mapping unit and testing platform.

The mapping unit developed in this work is illustrated in Figure 7a. Figure 7b shows the mounting method of the mapping unit on the testing platform. All system components are built with the Robotic Operating System (ROS) framework [45], which allows the intercommunication between sensors. An onboard computer with quad-cores running at 1.7 GHz and 4 GB of RAM is attached to the vehicle to host the proposed software system. The chassis of the robot is based on a three wheels differential drive model.

### 6.1. Evaluation of the Proposed Feature Selection Method

**Figure 8:**
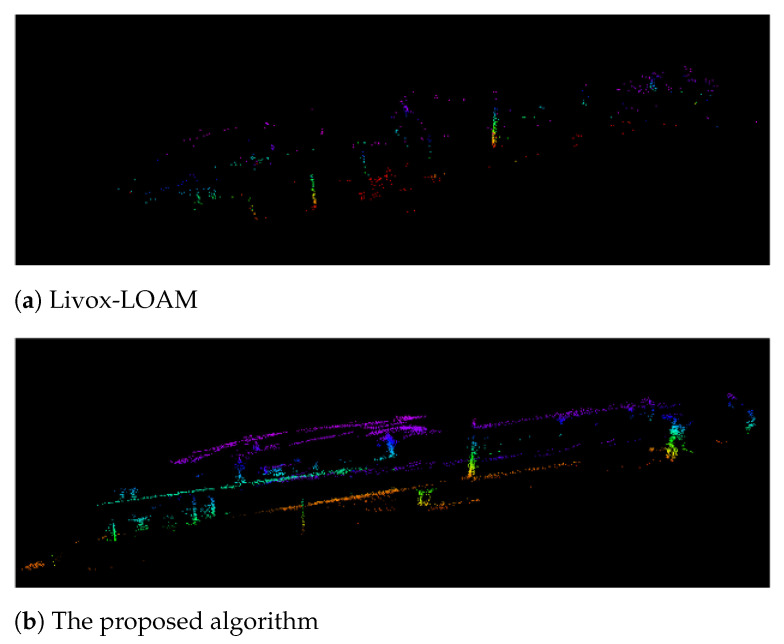
Compare corner feature collection between the proposed algorithm and Livox-LOAM in 10 scan frames.

The presented feature selection algorithm was evaluated with the ground platform travelling through a long corridor inside the Monash University Engineering Building. With the proposed feature selection method, the SLAM system is able to identify feature points in the environment more efficiently. Shown in Figure 8a, Livox-LOAM is less sensitive to corner features in the testing environment. The algorithm extracts a very limited amount of corner points from the building structure. Compare with Livox-LOAM, in Figure 8b, the proposed algorithm successfully covered a larger number of corner points. It is worth noting that the proposed system correctly identifies the corners between the floor, ceiling and the wall, which significantly improves the coverage of corner features.

**Figure 9:**
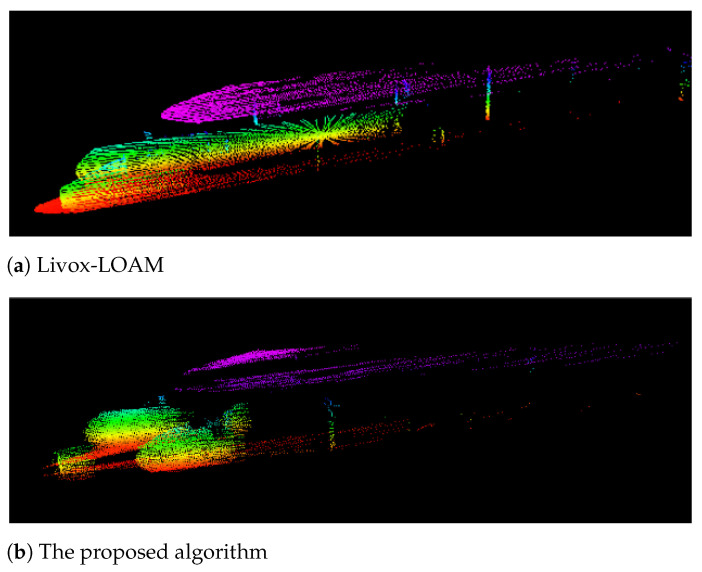
Comparison of plane feature collection between the proposed algorithm and Livox-LOAM in 10 scan frames.

On the other hand, Livox-LOAM classifies a vast amount of points as plane features. From Figure 9a, it could be seen that plane feature selection process include some non-plane points in the results. However, plane feature selection is more restricted with the proposed algorithm, where only five plane sections are considered in each scan. As a result, the proposed system only picks the five smoothest surfaces in the current scan frame as the plane feature.

To further investigate the feature selection difference between the proposed system and the Livox-LOAM, the numbers of feature points collected by both algorithms were compared in six different attempts. Each attempt is base on a single scan reading from a Livox Mid-40 running at 10 Hz. To average the result, this study took the tests in different environments, with indoor and outdoor scenarios. The results of the tests are shown in Table 2. Using Livox-LOAM, the mapping system only identified a limited amount of corner points in the environment. The unbalanced feature numbers lead the system to rely more on plane points than corner points.

With the proposed point preprocessing method, the system built a pre-knowledge about the scanning surface, which helps the system identify more corner features from the environment. Additionally, since feature selections are limited by sections, only the high-quality surfaces are considered as the plane feature in the proposed approach. Overall, the proposed feature selection algorithm can create a more accurate and balanced feature selection results for the following scan-matching process.

### 6.2. Evaluation by the Odometry Comparison

**Figure 10:**
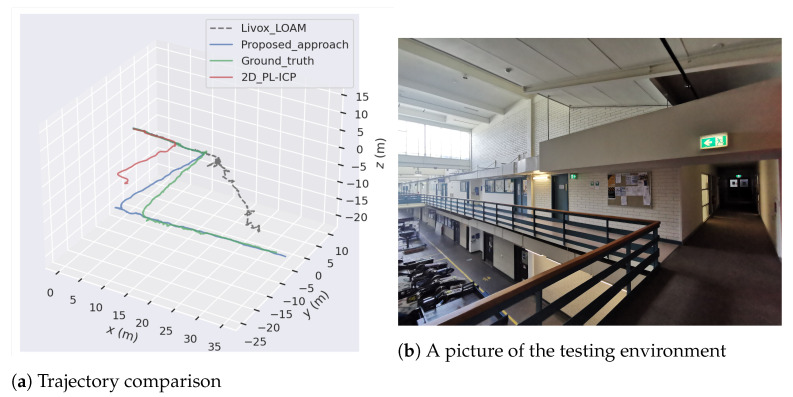
Trajectory Comparison between proposed approach, Livox-LOAM and the ground truth.

Using three corridors connected by two sharp turns, this section investigates the performance of the proposed odometry interpolation algorithm. During the experiments, the range of both LiDARs were limited to 30 m. The ground vehicle was travelling at around 0.95 m/s with an angular velocity of 1.17 rad/s while turning. A laser interferometer-based tracker was used in these experiments to record the ground truth of the system state. The laser tracker tracks the retroreflector mounted on the vehicle to record its six DOF motions. Figure 10a describes the trajectory comparison between the three DOF pose estimation from the 2D LiDAR, the six DOF pose estimation from 3D SSL with Livox-LOAM, the presented 2D-3D mixed SLAM approach and the ground truth collected by the laser tracker. The experiment environment is illustrated in Figure 10b, with the robot travelled from the right side of the image to the left.

**Figure 11:**
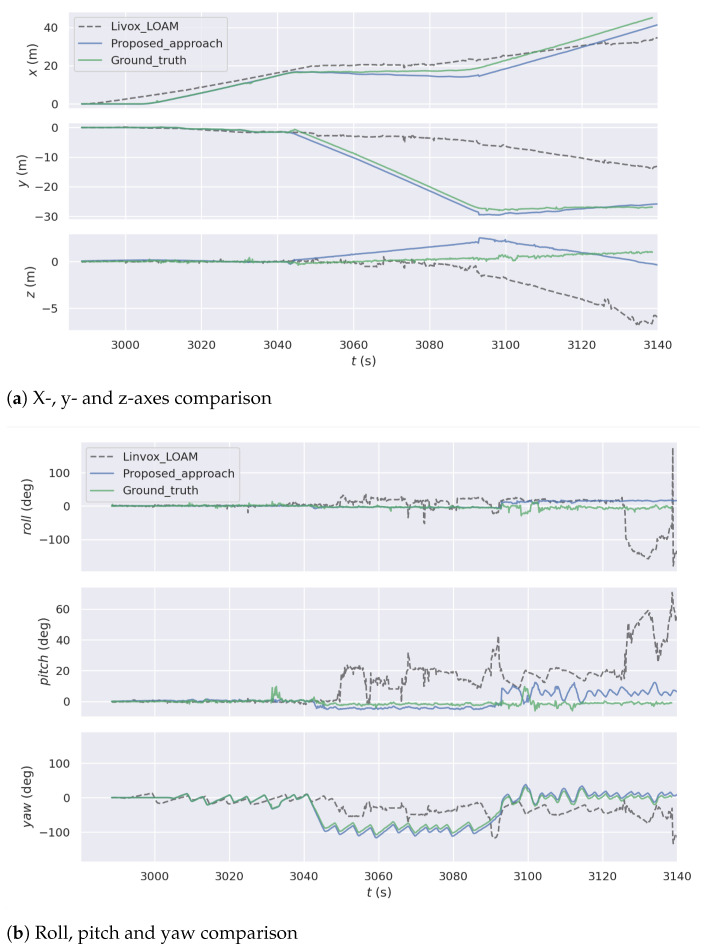
Odometry Comparison between proposed approach, Livox-LOAM and the ground truth.

In Figure 10a, the outcome of 2D LiDAR incremental pose estimation illustrates its outstanding performance in corners. However, with PL-ICP algorithm, the three DOF odometry is vulnerable to feature less long corridors. On the other hand, six DOF pose estimation using Livox-LOAM successfully positioned the system in the long corridor scenario during the first one-third of the test. Nevertheless, as shown in Figure 10a, with limited FOV, the system has poor performance in sharp turns, especially when obstacles are close to the LiDAR. The error accumulated on the map which affected future mapping results and caused large drifts in the trajectory. The trajectory of the proposed algorithm is the closest to the ground truth in the experiments. The LiDAR odometry performance significantly improved with the proposed system, where the displacement between system trajectory and the ground truth is minimised. An axis-wise comparison between the proposed system, the Livox-LOAM and the ground truth is illustrated in Figure 11a. The proposed odometry interpolation method successfully enhanced the robustness in x- and y- axes. Similar results can be seen in Figure 11b, where the proposed system significantly outperforms the compared approach in motions on yaw axis. It is worth to note that compared with the ground truth the proposed system has less accuracy on z- roll and pitch axes as they are not enhanced by the three DOF odometry interpolation method described in Section 5. However, the presented approach still outperforms the Livox-LOAM algorithm with the dual-LiDAR feature extraction method described in Section 4.

Compared with the ground truth, the proposed approach has absolute position error (APE) of 5.64. Since the accumulated mapping error of the Livox-LOAM approach is significantly larger, its APE in the same test was 41.32. Additionally, on the average, the proposed algorithm is able to utilize 12 times more corner feature points than the Livox-LOAM.

### 6.3. Evaluation by Mapping Result

Experiments were conducted around the Monash University campus to further investigate the proposed system’s performance compared with the Livox-LOAM. These experiments were designed in scenarios that could potentially receive different results from the two algorithms.

**Figure 12:**
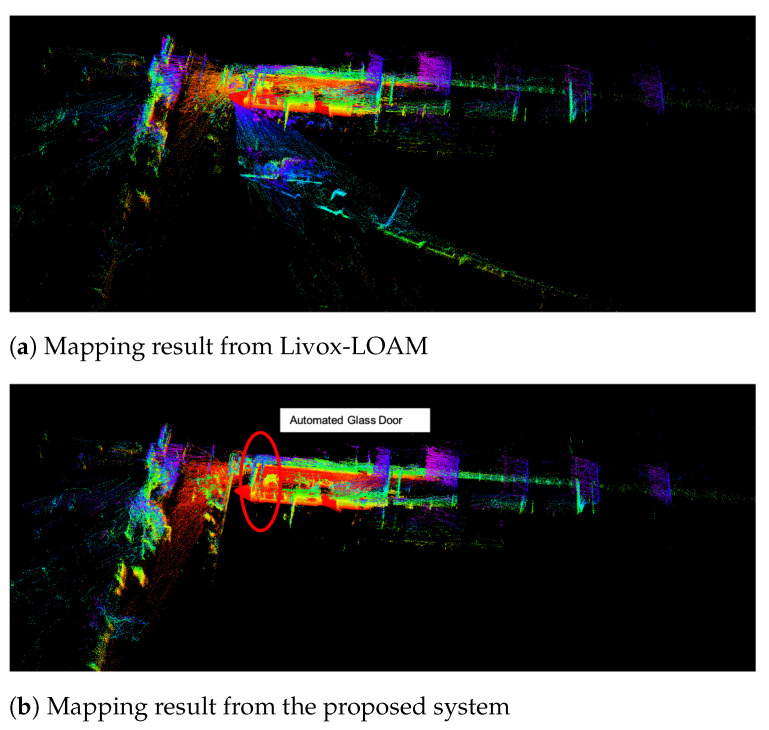
Mapping through an automated glass door.

Figure 12 demonstrates the experiment results of the testing platform travelling through an automated glass door. While the robot was approaching the door, both side of the door opens towards the mapping system. Using Livox-LOAM, even the algorithm is able to receive the majority of the readings through the glass, the moving door still caused significant mismatches, which results in the mapping error on Figure 12a. In the same test, the mapping result from the proposed system (Figure 12b) shows a noticeable improvement as no significant errors are recorded on the map.

**Figure 13:**
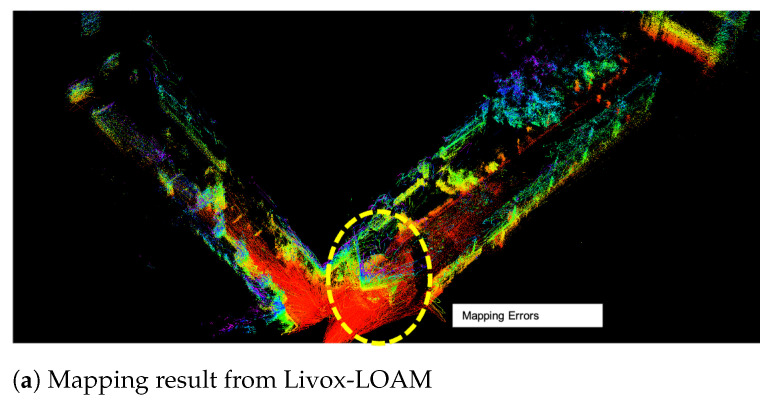

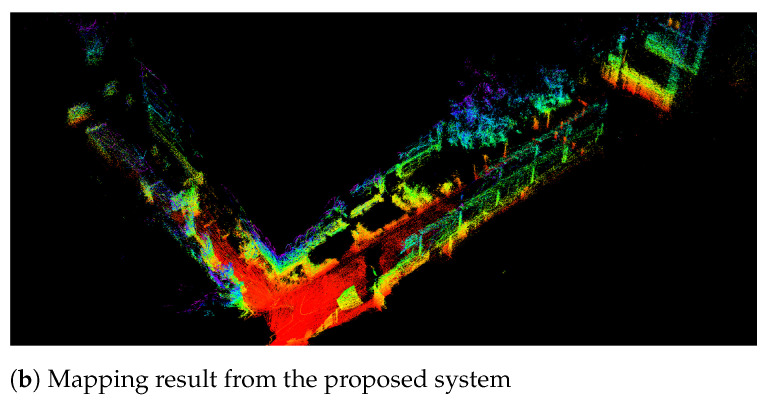
Mapping results of a sharp hook turn action.

A pair of sharp hook turn tests were conducted to investigate the performance of the proposed system, especially its odometry stability. From the results illustrated in Figure 13, it can observed that the improvement of the proposed method over the Livox-LOAM is significant. From observation, single SSL mapping is vulnerable to sizeable obstacles which occupying a large proportion of its FOV. In the test, while the robot is turning, it moves towards a large and featureless wall, which introduces error to the matching function.

## 7. Conclusions and Discussion

This paper described a hybrid LiDAR mapping model which uses a 2D spinning LiDAR and a 3D SSL to perform six DOF pose estimation and mapping tasks. The proposed system features a point cloud preprocessing mechanism and a three DOF interpolated six DOF odometry. The system developed in this work inherits the structure of LOAM and Livox-LOAM algorithms. In addition, the developed approach strengthen the robustness of the inherited methods with a supplemental 2D LiDAR. With the conducted experiments, this work proves its capability to improve the stability of a SSL mapping system by utilising 12 times more corner feature points. The proposed algorithm only recorded an APE of 5.64 in the experiment, which is significantly improved compare with the Livox-LOAM system. However, this comparison is limited as the proposed system uses an extra 2D LiDAR than the Livox-LOAM system. In addition, enhancing the corner feature selection through FOV segmentation restricted the system’s capability of selecting plane features. Moreover, the system developed in this work takes advantage of urban terrain features. The effectiveness of applying this system to other terrains is still unstudied. Furthermore, the implemented dual-LiDAR pose estimation methodology does not take into account the motions in the z-axis, pitch or roll. Improving the system performance in these three axes requires further study. 

## Figures and Tables

**Table 1:** Performance comparison between Hokuyo UST-20LX, Livox Mid-40 and Velodyne HDL-64E.

	Channels	Range (Up to)	Rotation Rate	Horizontal FOV	Vertical FOV	Angular Resolution	Accuracy
Hokuyo UST-20LX	1	60 m	43,240 pts/s	270°	N/A	0.25°	±40 mm
Livox Mid-40	1	260 m	100,000 pts/s	38.4°	38.4°	0.05°	±2 cm
Velodyne HDL-64E	64	120 m	1,300,000 pts/s	360°	26.9°	0.08°	±2 cm

**Table 2:** Number of different feature points selected by Livox-LOAM and the proposed algorithm in 1 frame of Livox Mid-40 scan reading with the sensor running at 10 Hz.

	Livox-LOAM		Proposed Algorithm	
	Corner	Plane	Corner	Plane
test_1_Indoor	15	3544	209	1544
test_2_Outdoor	11	1945	150	1325
test_3_Indoor	9	3064	174	2004
test_4_Outdoor	32	2931	95	1754
test_5_Indoor	17	2815	357	1388
test_6_Indoor	4	2032	235	2084
